# Impact of long‐term chromosomal shuffling on the multispecies coalescent analysis of two anthropoid primate lineages

**DOI:** 10.1002/ece3.3736

**Published:** 2017-12-20

**Authors:** Carlos G. Schrago, Beatriz Mello, Anieli G. Pereira, Carolina Furtado, Hector N. Seuánez

**Affiliations:** ^1^ Department of Genetics Federal University of Rio de Janeiro Rio de Janeiro RJ Brazil; ^2^ Division of Genetics National Cancer Institute Rio de Janeiro Brazil

**Keywords:** divergence times, karyotypic evolution, multispecies coalescent, recombination, species tree

## Abstract

Multispecies coalescent (MSC) theory assumes that gene trees inferred from individual loci are independent trials of the MSC process. As genes might be physically close in syntenic associations spanning along chromosome regions, these assumptions might be flawed in evolutionary lineages with substantial karyotypic shuffling. Neotropical primates (NP) represent an ideal case for assessing the performance of MSC methods in such scenarios because chromosome diploid number varies significantly in this lineage. To this end, we investigated the effect of sequence length on the theoretical expectations of MSC model, as well as the results of coalescent‐based tree inference methods. This was carried out by comparing NP with hominids, a lineage in which chromosome macrostructure has been stable for at least 15 million years. We found that departure from the MSC model in Neotropical primates decreased with smaller sequence fragments, where sites sharing the same evolutionary history were more frequently found than in longer fragments. This scenario probably resulted from extensive karyotypic rearrangement occurring during the radiation of NP, contrary to the comparatively stable chromosome evolution in hominids.

## 1 INTRODUCTION

Phylogenetic analysis has been substantially impacted in the last decade by the development of methods for inferring species trees from gene trees or gene genealogies (Edwards, 2009; Liu, Yu, Kubatko, Pearl, & Edwards, 2009a; Maddison, 1997). This impact was mostly due to the development of multispecies coalescence (MSC) theory, which explicitly models the distribution of gene trees within a given species tree (Degnan & Salter, 2005; Liu, Yu, & Edwards, 2010; Liu, Yu, Pearl, & Edwards, 2009b). It has been argued that MSC methods have effectively unveiled the evolutionary relationships of long‐standing phylogenetic puzzles, like the early diversification of placental mammals (McCormack et al., 2012; Song, Liu, Edwards, & Wu, 2012) and the phylogeny of bird orders (Jarvis et al., 2014). MSC relies on the assumption of complete independence of loci for estimating the distribution of gene trees, which are considered to be independent trials of the MSC process (Liu et al., 2009a).

Recently, Springer and Gatesy (2016) argued that for lineages as old as placental mammals, genomic segments free from intralocus recombination were limited to 12 bp. Thus, as MSC analyses frequently rely on loci encompassing thousands of base pairs (Song et al., 2012), lack of intralocus recombination was expected to be highly unlikely, a reason why a “concatalescence” analysis would frequently be performed, instead of the standard MSC. Lanier and Knowles (2012) conducted an extensive evaluation of the consequences of recombination on the performance of MSC methods and found that only when incomplete lineage sorting (ILS) was very high, inference of species trees was affected by recombination.

Problems related to the lack of loci independence and the MSC model should be ideally investigated in extensively studied empirical cases. In this respect, primates are an ideal taxon because they comprise a well‐studied lineage with available genome data, which makes the scrutiny of MSC feasible. For instance, the evolutionary affinities of great apes and humans have been extensively investigated using coalescent analyses (Burgess & Yang, 2008; Chen & Li, 2001; Hobolth, Christensen, Mailund, & Schierup, 2007; Schrago, 2014a). These species share remarkable karyotypic similarities, or interspecific chromosome “homoeologies,” because of their similar chromosome diploid number (2*n* = 46 in man; 2*n* = 48 in the great apes) and their macrostructural karyotypic attributes (chromosome banding patterns), which were largely conserved for more than 15 Ma (Seuanez, 1979; Yunis & Prakash, 1982).

Conversely, the chromosome complement of Neotropical primates (NP) underwent a dramatic karyotypic shuffling during the evolutionary divergence of these species (Stanyon et al., 2008), which took approximately 40 million years (Perelman et al., 2011; Schrago, 2007). Differently from hominids, chromosome diploid number varies sharply in NP, ranging from 16 in *Callicebus lugens* (Bonvicino et al., 2003) to 62 in *Brachyteles arachnoides* and *Lagothrix lagotricha*. Moreover, the phylogeny of the major NP lineages has been controversial as early studies employing molecular data (Schneider et al., 1993; Wildman, Jameson, Opazo, & Yi, 2009). Recently, Schrago, Menezes, Furtado, Bonvicino, and Seuanez (2014) carried out MSC analyses of NP and showed a significant discordance between the distribution of mismatch gene tree topologies and the species tree. Authors used data from the partially sequenced genomes of *C. lugens* and *B. arachnoides*, two species accounting for the most extreme differences in diploid chromosome number within NP. Using a four‐species phylogeny (rooted with *Homo sapiens*), which included a representative of each NP family, namely Atelidae (*B. arachnoides*), Pitheciidae (*C. lugens*), and Cebidae (*Callithrix jacchus*), the gene tree topology matching the NP species tree, that is, *Brachyteles*/*Callithrix*, accounted for 47% of the trees from >25,000 analyzed loci. This low frequency of species tree/gene tree topological match resulted from the combined effects of a rapid speciation process, reflected by short internal branches and a large, long‐term, ancestral effective population size (>500,000 Wright‐Fisher individuals).

Moreover, differently from theoretical expectations, the frequencies of the two gene topologies mismatching the species tree were significantly unbalanced because the *Callithrix*/*Callicebus* and the *Brachyteles*/*Callicebus* topologies were recovered in 29% and 24% of gene trees, respectively. Schrago et al. (2014) hypothesized that this imbalance was due to substantial karyotypic differences, which violated the standard MSC model. On the other hand, the distribution of mismatched gene trees for the/human/chimp/gorilla trio, rooted with orangutan (Hominidae), fitted accordingly to theoretical expectations, with *Homo*/*Gorilla* and *Pan*/*Gorilla* topologies accounting for ~12% of gene trees. As the rate of chromosome evolution in hominids was relatively low, we may assume that the physical distribution of loci was kept stable along their diversification. For example, because of this lower evolutionary chromosome rate, a locus in human chromosome 1 and its hominid homoeologues was maintained historically independent of another locus in human chromosome 3 and its hominid homoeologues for more than 15 million years. The same cannot be said of NP, because of intense chromosomal shuffling. Therefore, mapping independent sites along the genome is easier in hominids.

The evolution of the major NP lineages is thus a compelling case for evaluating the effects of long‐term chromosomal shuffling on MSC analysis, because a higher amount of karyotypic change may violate the assumption of independence between loci during the evolutionary time. Using genomic data, we performed a comparative analysis of NP and hominids to investigate the effects of sequence length on the theoretical predictions of the MSC model using four‐species compositions. Finally, we were also able to investigate the impact of karyotypic evolution on the performance of two competing approaches in phylogenomics, concatenation, and phylogeny inference based on MSC theory.

## 2 MATERIALS AND METHODS

To evaluate the relative effect of chromosomal shuffling on the MSC, we used two datasets of anthropoid primate lineages with contrasting rates of chromosomal evolution, namely Neotropical primates (high rate) and the great apes (low rate). For NP, we employed the dataset of Schrago et al. (2014), consisting of 25,955 orthologous loci, with 3,500 bp on average, from *Brachyteles arachnoides* (Atelidae), *Callicebus lugens* (Pitheciidae), and *Callithrix jacchus* (Cebidae), rooted with *Homo sapiens*. Hominid (great apes) data were the same as previously used by Schrago (2014b), consisting of 15,744 orthologous 5,000‐bp fragments randomly sampled across chromosome alignments of *Homo sapiens*,* Pan troglodytes*,* Gorilla gorilla*, and *Pongo abelii* (outgroup), as available in ensembl compara repository (ftp://ftp.ensembl.org/pub/release-67/emf/ensembl-compara).

**Figure 1:**
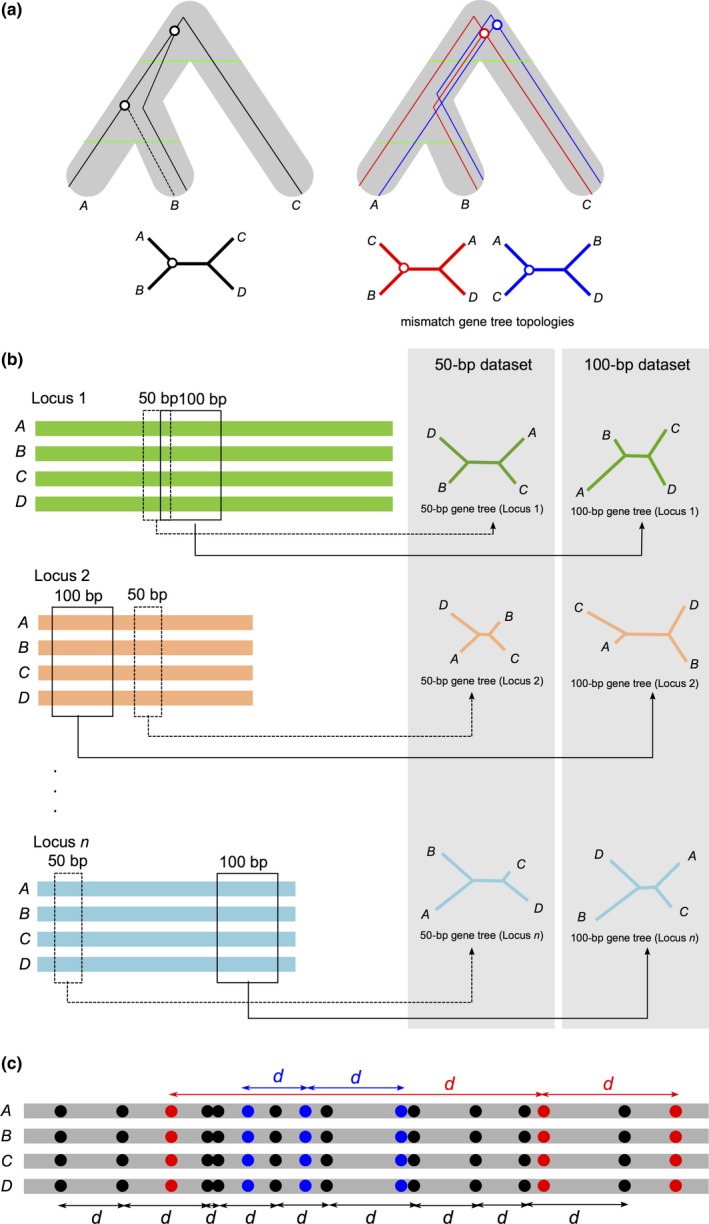
Schematic representation of the methodology employed to investigate the impact of long‐term chromosomal shuffling on the MSC model. (a) Possible gene tree topologies for a given species tree (shaded area). Matching gene tree topology is shown in the left. In this tree, coalescent events between a pair of alleles may occur either in the ancestral (A,B) population or in the ancestor of ((A,B),C). On the other hand, coalescent events of mismatch gene tree topologies (right) will take place in the ancestral ((A, B),C) population. Coalescent times of the alleles of the two lineages that consist the most inclusive clade of the gene tree are shown in solid white circles. Solid green lines depict the ages of genetic isolation of species. (b) Sequence fragments of each length class were randomly collected from the loci in both NP and hominids. Those fragments were then subjected to phylogenetic analysis in both PhyML and MrBayes. Gene tree topologies of each length class were used to run ASTRAL. (c) Distribution of PI sites along loci. PI sites supporting the species tree are shown in black, whereas PI sites for mismatch gene tree topologies are depicted using the color scheme in (a). As a given PI site increase in frequency, distances between those sites decrease

The independence of molecular markers was analyzed using a methodological approach for creating presumably evolutionarily unlinked genomic regions. Theoretically, the longer a sequence, the lower its probability of containing sites with coincidental evolutionary histories, thus violating MSC model. We expect that, as sequence length increases, the probability of violating MSC assumptions also increases because of a higher probability of analyzing sites with different evolutionary histories. In hominids, because of chromosomal stability, the effect of model violation will be shared by both mismatch topologies, whereas the same cannot be said of NP. Figure 1a summarizes MSC model expectations, showing how mismatch gene trees are recovered if alleles fail to coalesce in the ancestral population of the most inclusive species tree clade, that is, *Brachyteles*/*Callithrix* in NP and *Homo*/*Pan* in hominids. Thus, the distribution of gene trees was obtained using sequence fragments of seven increasing length classes (50, 100, 200, 500, 1,000, 1,500, and 2,000 bp). For each length class, datasets were created by randomly selecting regions from the original loci (Figure 1b). Samplings of sequence fragments were performed without replacement, that is, each locus was sampled only once for each length class.

Departure from the standard MSC model was analyzed based on the theoretical prediction that the frequency of both mismatch gene tree topologies is expected to be equal in four‐species phylogenies. For instance, rooting with *Pongo*, if ((*Homo*,* Pan*), *Gorilla*) is the species tree, the frequencies of mismatch gene tree topologies, that is, ((*Gorilla*,* Pan*), *Homo*) and ((*Homo*,* Gorilla*), *Pan*), are expected to be equivalent under complete independence of gene loci (Pamilo & Nei, 1988). If chromosomal shuffling is an issue for MSC analysis of NP, we hypothesized that differences between the frequencies of mismatch topologies (i.e., frequency imbalance) would decrease with decreasing fragment length, because the shorter the fragments, the higher their probability of containing nucleotide sites sharing the same evolutionary history, thus behaving closely to MSC expectations. Moreover, coalescent times, measured by the lengths of terminal branches, should be equal for both mismatch gene trees. Reduced fragment length, however, would also increase the stochastic error of phylogenetic inference, a reason why the topological support of inferred gene trees was also evaluated, therefore ensuring that topologies were reasonably estimated.

### 2.1 Phylogenetic analysis

Gene tree topologies were estimated using both maximum likelihood (ML) and Bayesian inference (BI) frameworks. ML analysis was carried out on PhyML 3 (Guindon et al., 2010) using the GTR + G4 model, whereas BI was implemented in MrBayes 3.2 (Ronquist & Huelsenbeck, 2003). In BI, two independent Markov chain Monte Carlo runs with four chains each were employed, and chains were sampled every 500th generation for a total of 1,000,000 cycles. The model of sequence evolution was standardized throughout ML and BI analyses. Topological support was estimated with the aLRT statistic (Anisimova & Gascuel, 2006) in ML and posterior clade probability in BI gene trees. Species tree reconstructions were run on ASTRAL (Mirarab et al., 2014). For the sake of comparison, we have also inferred topologies from alignments obtained concatenating sequences from each fragment length class.

### 2.2 Distribution of phylogenetically informative sites along biological sequences

As an additional criterion to evaluate the impact of chromosomal shuffling on the phylogenetic signal present in the alignments of both anthropoid primate lineages, we investigated the distribution of phylogenetically informative (PI) sites along loci. In four‐species alignments, the nucleotide composition of a PI site is that of the “1100” type, that is, a site containing one nucleotide type shared by any two species and another nucleotide type shared by the two remaining species, allowing for parsimonious discrimination of three possible unrooted tree topologies. If PI sites for each possible topology were equally frequent across the genome, we expected that reduction in sequence length would increase the probability of random inference of gene tree topologies. Moreover, if PI sites were equally frequent along genomes, the average distance (in bp) between a pair of adjacent PI sites supporting the same topology would be equivalent for the three possible unrooted tree topologies. On the other hand, the larger the number of PI sites in genome alignments supporting one of the three possible unrooted topologies, the smaller will be the average distance between two adjacent sites supporting that same tree topology (Figure 1c).

## 3 RESULTS

**Figure 2:**
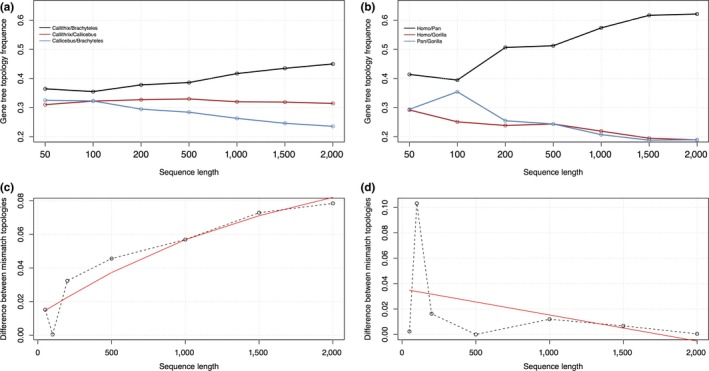
Distribution of gene tree ML topologies with respect to sequence length (a) Neotropical primates; (b) Hominids. The *Callithrix*/*Brachyteles* (a) and *Homo*/*Pan* (b) topologies match the species tree. Trends of differences between frequencies of mismatch topologies are shown in NP (c) and Hominids (d)

**Figure 3:**
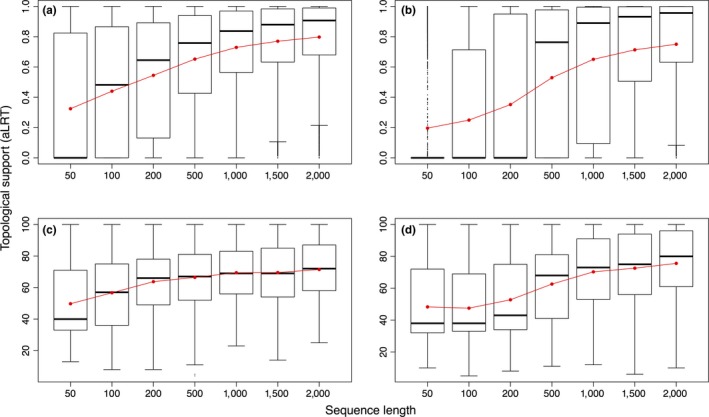
Topological support for the correct ML gene tree obtained with sequences of different length. (a and b) aLRT statistics for *Callithrix*/*Brachyteles* and *Homo*/*Pan*, respectively. (c and d) Posterior probabilities of *Callithrix*/*Brachyteles* and *Homo*/*Pan* tree topologies, respectively

We found that the relative frequencies of gene tree topologies were significantly altered with respect to fragment length (Figure 2). In both anthropoid primate datasets, the gene tree topology matching the species tree was the most frequent topology independently of fragment length and its relative frequency increased with longer sequences. For instance, with 200‐bp fragments, the *Callithrix*/*Brachyteles* topology accounted for 36.4% of gene trees while this frequency shifted to 45.0% with 2,000 bp. A similar pattern was found for the *Homo*/*Pan* topology, changing from 51.4% (with 200 bp) to 62.1% (with 2,000 bp). Frequencies of mismatch gene tree topologies responded differently in both primate datasets. In NP, the *Callithrix*/*Callicebus* topology accounted for 31.4% of gene trees obtained from 2,000‐bp fragments, whereas the *Brachyteles/Callicebus* topology for 23.6%. Moreover, in NP, differences between frequencies of mismatch topologies steadily increased with increasing fragment length. Conversely, in hominids, the frequencies of both mismatch gene tree topologies differed when <200‐bp fragments were analyzed, but then remained rather constant at ca. 18% with 2,000 bp. In hominids, differences between the frequencies of mismatch topologies were negligible with length nearly 2,000 bp (Figure 2). The average support for gene trees matching the species tree also increased with increasing fragment length (Figure 3).

**Figure 4:**
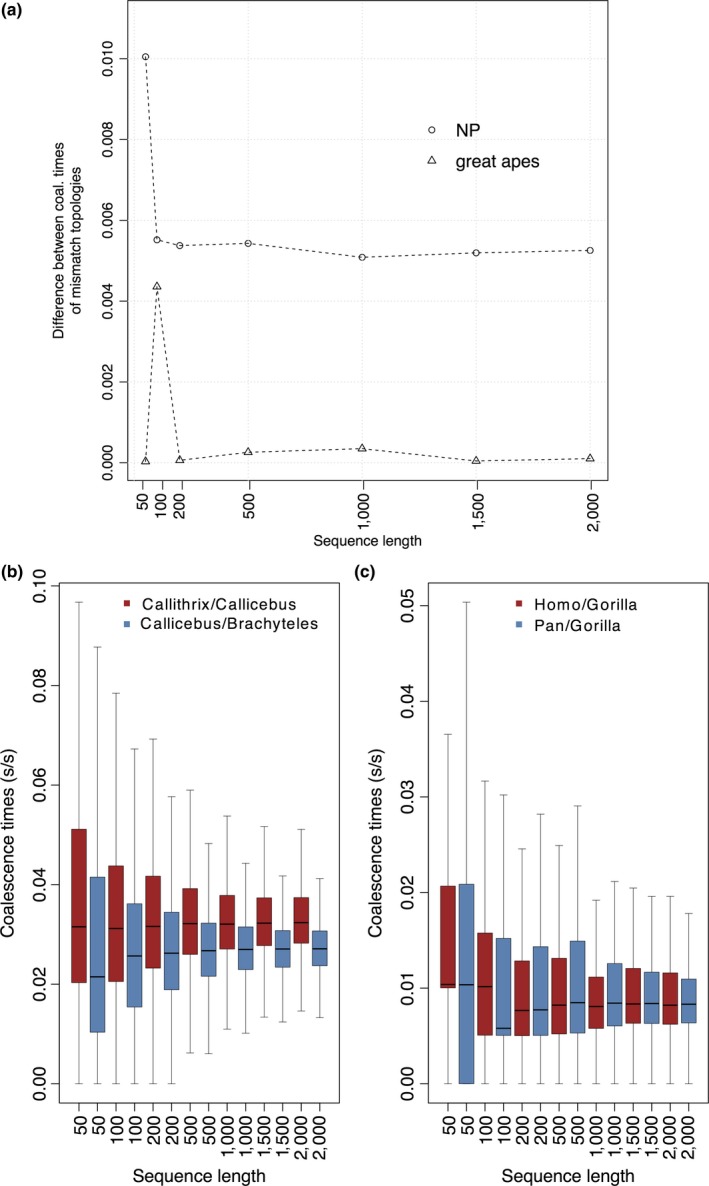
Coalescence times of mismatch topologies. Difference between average coalescence times of mismatch topologies with respect to sequence length (a). Distribution of coalescence times of NP (b) and Hominids (c) mismatch topologies

The distribution of differences in coalescence times of the most inclusive clades between mismatch topologies also showed different trends in both primate datasets when fragment length increased (Figure 4). In NP, in datasets with >200‐bp fragments, difference between mean coalescent times of mismatch topologies was equivalent, although not null, reaching approximately 0.005 substitutions/site. The same difference was nearly null in hominids, accordingly to theoretical expectations (Figure 4a). In both scenarios, the variance estimates of coalescent times of mismatch topologies also tended to be stable with increasing fragment length (Figure 4b,c).

**Figure 5:**
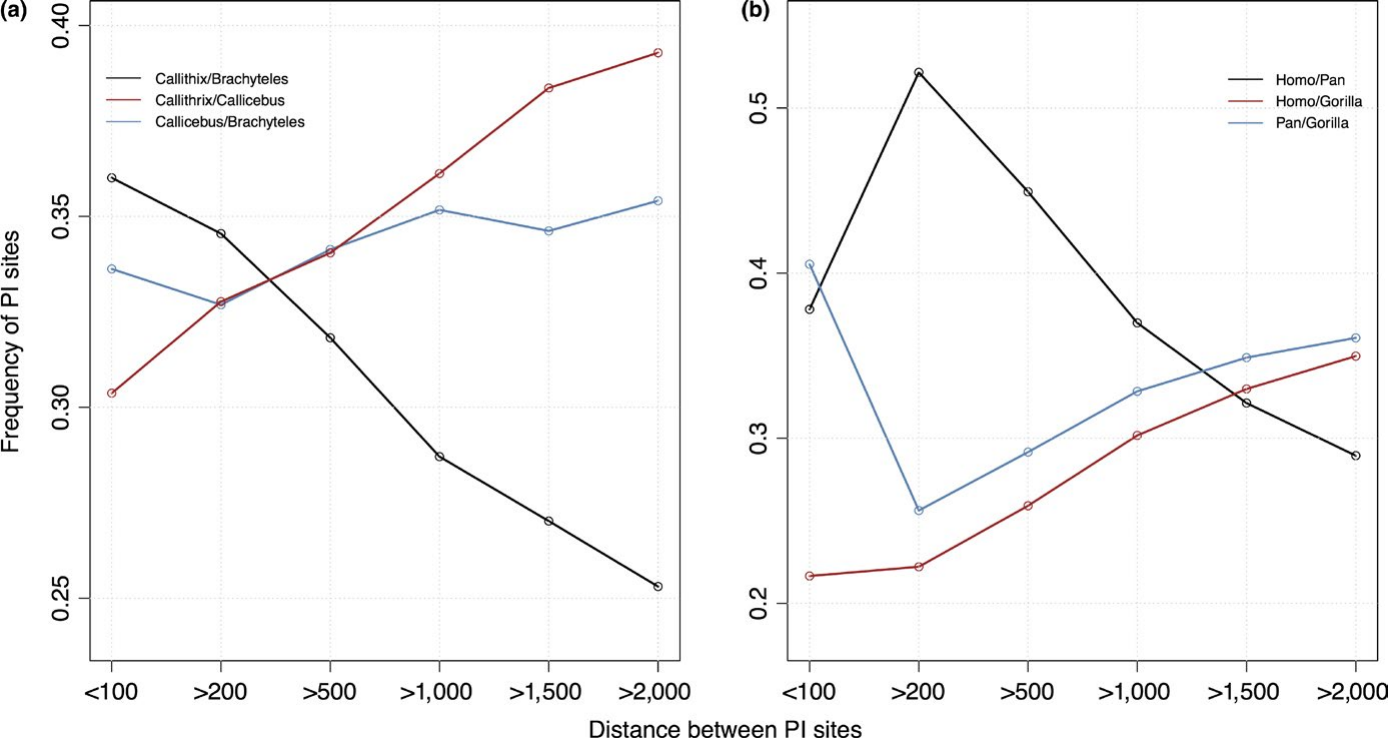
Frequency of phylogenetically informative sites for each topological arrangement separated by different distances between adjacent PI sites along genomic segments in (a) NP and (b) Hominids

The total number of PI sites supporting the species tree topology was greater than the total number of mismatching sites in both primate datasets. Consequently, distances between adjacent PI sites supporting the species tree topology are expected to be comparatively smaller (Figure 5). When tracking the frequency of PI sites separated by larger distances, those supporting the species tree were rarely sampled, while those supporting mismatch topologies became more frequent. In hominids, the frequencies of PI sites separated by >2,000 bp supporting both *Homo*/*Gorilla* and *Pan*/*Gorilla* mismatch topologies were equivalent (~33%). In NP, contrary to theoretical expectations, the frequency of PI sites supporting the *Callithrix*/*Callicebus* mismatch topology was higher than the *Brachyteles*/*Callicebus* topology with increasing between‐PI site distances. In NP, the frequency of PI sites supporting the species tree, and that were separated by larger distances, steadily decreased at a faster rate when compared to hominids (Figure 5). For instance, in NP, most PI sites separated by >500 bp supported mismatch topologies, whereas, in hominids, the same pattern occurred with sites separated by >1,500 bp.

Although the distribution of mismatch topologies presented different trends in both primate datasets, all heuristic MSC methods for estimating species trees correctly inferred NP and hominid phylogenies independent of fragment length class. The topological support associated with the inferred species trees also increased with increasing fragment length, providing evidence of the statistical consistency of these methods. In NP, for instance, the statistical support for species trees estimated in ASTRAL shifted from 58% to 100% in the datasets containing 50‐ and 1,000‐bp sequence fragments, respectively. In datasets with sequences longer than 1,000 bp, topological support was maximized in all length classes.

When sequences of each length class were concatenated, topologies were correctly inferred in all cases with both ML and BI. The correct topology was inferred even by concatenating short 50‐bp sequences, achieving maximum statistical support in both NP and hominid datasets; this scenario was replicated for all classes of sequence length. Concatenation, therefore, also showed statistical consistency because increasing fragment length reduced the uncertainty of inferred true topology.

## 4 DISCUSSION

**Figure 6:**
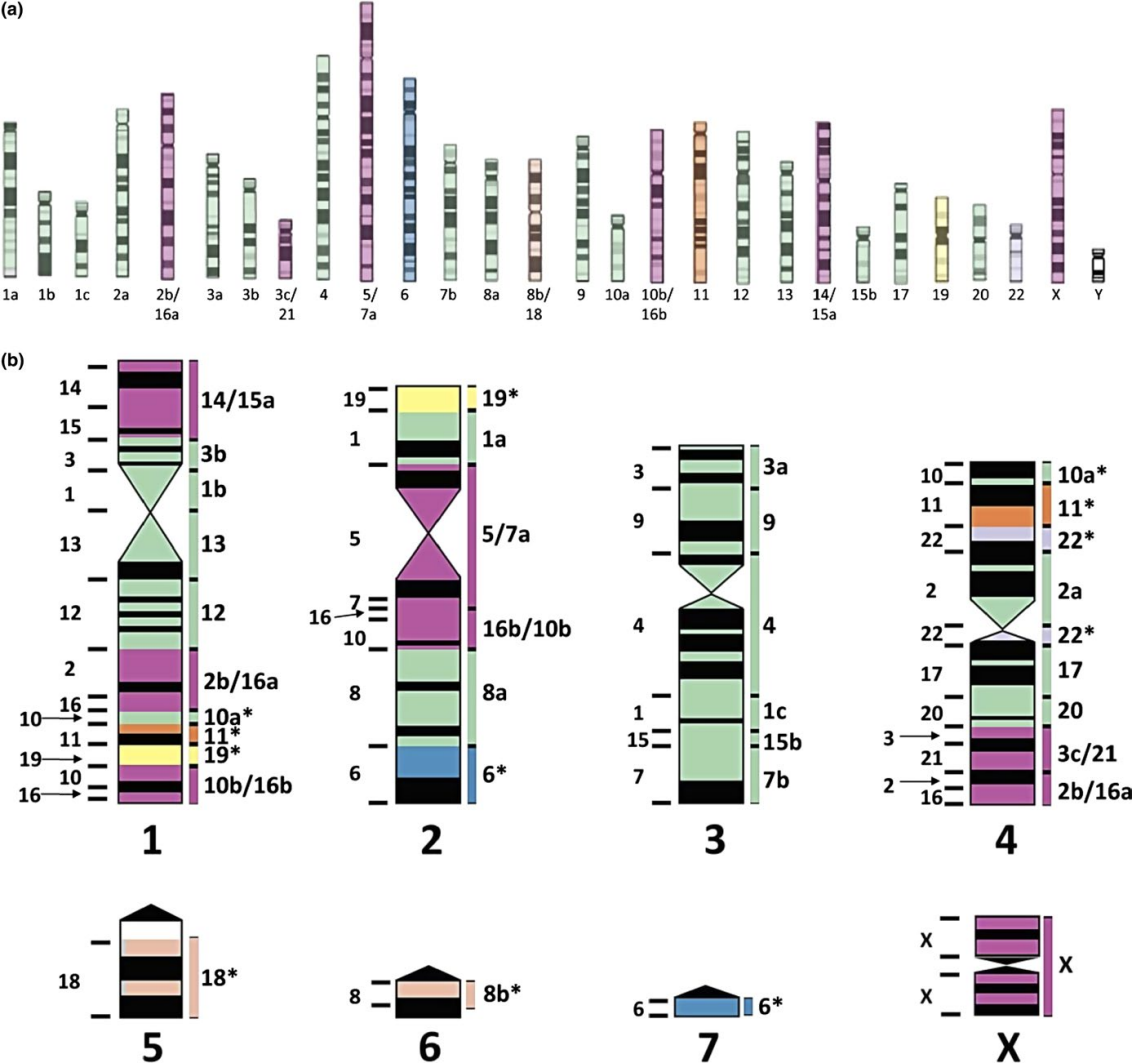
Karyotypic evolution of NP. (a) Ideogram of the presumptive ancestral karyotype of Neotropical primates (NP). Numbers refer to human chromosome probes; letters indicate disruptions respective to human syntenies. Different colors indicate which ancestral chromosomes are present in *Callicebus lugens* as an ancestral syntenic association (red), de novo, non‐rearranged ancestral syntenies fused to other non‐rearranged ancestral syntenies (green) and disrupted ancestral syntenies (blue, brown, orange, yellow, and gray, corresponding to 6, 8b/18, 11, 19, and 22, respectively). (b) Ideogram of *Callicebus lugens* chromosomes (2*n* = 16). Regions painted with human chromosome probes are shown on the left of each chromosome. Regions corresponding to ancestral NP chromosomes are shown on the right. Colored areas indicate the presumed origin of each region respective to the ancestral NP karyotype. Asterisks indicate fission products. Modified idiograms, from Stanyon et al. (2003)

We have shown that distributions of gene tree topologies differed in two anthropoid primate lineages whose evolutionary radiations occurred with strikingly different levels of chromosomal rearrangement. In NP, the difference between frequencies of the two mismatch topologies, used here as a proxy for assessing the standard MSC model, decreased with shorter fragments. This outcome could be understood by proposing that shorter sequences increased the probability of obtaining evolutionary independent loci in genomes subjected to extensive chromosomal shuffling, such as NP. For instance, in *Callicebus lugens*, with a diploid number of 16 chromosomes (Bonvicino et al., 2003), genomic contraction has been prominent, with most human chromosome probes painting four large chromosome pairs (Stanyon, Bonvicino, Svartman, & Seuanez, 2003). This extreme level of karyotypic rearrangement might be explained by duplication of two ancestral NP syntenies (2b/16a and 10b/16b), six fissions, one inversion, and 31 fusion events for deriving the karyotype of *Callicebus lugens* from the presumed ancestral NP karyotype with 2*n* = 54 (Figure 6). Conversely, a similar derivation of the karyotype of *Brachyteles arachnoides* (2*n* = 62) requires seven rearrangements (four fissions, one 10/16 duplication, one inversion, and one fusion) while nine autosomal syntenies and the X chromosome were found to be evolutionarily conserved (de Oliveira et al., 2005). Moreover, the karyotype of *Callithrix jacchus* (Sherlock, Griffin, Delhanty, & Parrington, 1996) showed 13 relict syntenies, while seven fusions and four fissions were required for its derivation from the presumed ancestral karyotype of all NP.

On the other hand, in hominids, in which karyotypic rearrangements have been less drastic, genome segments sharing the same evolutionary history can be easily sampled across different classes of fragment length. Thus, increasing fragment length would not challenge the standard MSC model, as long as each fragment is statistically independent. The observation that increasing sequence length decreases the probability of sampling sites with coincidental histories was criticized by Springer and Gatesy (2016) as a “full circle” return to the early stages of molecular phylogenetics, when DNA sequencing was technically challenging and expensive. Our empirical results can be used to evaluate the “full circle” criticism. In both datasets analyzed, independently of sequence length, heuristic MSC methods and traditional concatenation all performed well. Both methods were statistically consistent even in cases where the distribution of gene tree topologies was anomalous (e.g., with 100 bp). In this sense, MSC methods are preferable if the researcher wishes to adopt an inferential framework nested within the foundations of population genetics, allowing for a theoretical unity between different areas of evolutionary genetics (Edwards et al., 2016; Lynch, 2007). Moreover, the efficacy of both MSC and concatenation methods in estimating the species tree, even with gene fragments as small as 200 bp, is operationally relevant because current genome sequencing technologies frequently produce short sequence reads that, according to our analysis, could be readily used in phylogenetic inference.

Although heuristic MSC methods were robust for topological inference of the species tree, estimation of population‐level parameters using standard MSC theoretical predictions was not uniform under different sequence lengths. Variation in the relative proportion of gene tree topologies would result in significantly different estimates of the length (in coalescent units) of the branch connecting the crown NP node and the *Callithrix*/*Brachyteles* split. The same is true for hominids. For instance, using the equation of theoretical probability of a topological match between gene trees and the species tree (Pamilo & Nei, 1988), this internode interval varied from 0.03 to 0.19 coalescent units in NP and 0.09 to 0.57 in hominids. These values contrasted with previous estimates of ~0.3 for NP (Schrago et al., 2014) and ~1.1 coalescent units for hominids (Hobolth et al., 2007; Schrago, 2014a). Thus, MSC theory should be used cautiously to estimate population parameters to investigate the action of evolutionary forces on ancestral species.

Because topological support also dropped with shorter sequence fragments (Figure 3), it might be argued that reduction in the difference between frequencies of mismatch topologies was caused by poor phylogenetic precision, instead of a better fit of the data to the MSC model. In the absence of phylogenetic information, the topological distribution of the three possible gene tree topologies should be approximately equivalent, making any difference between topology frequencies close to zero. This explanation is unlikely because the relative frequency of the gene trees matching the species tree was always higher, and the NP pattern was not replicated in hominids. In hominids, with 100‐bp sequence fragments, the difference between the frequencies of mismatch topologies was higher, contrary to the argument of similar frequencies resulting from a reduced phylogenetic signal in NP.

Aside from the physical dependence of loci, deviation from the MSC model is also expected by the action of natural selection (Degnan & Rosenberg, 2009; Liu et al., 2009a). For instance, if the tempo and mode of molecular evolution varied significantly in *Callithrix*,* Callicebus*, and *Brachyteles*, evolutionary convergence or parallelism would deviate the distribution of mismatch topologies in a manner similar to long branch attraction, although MSC was found to be robust in this scenario (Liu, Xi, & Davis, 2015). This would be a problem even if sequences failed to reject the molecular clock (equal rates, different modes). We argue, though, that selection is not the likeliest explanation for the deviation found in the frequency of mismatch topologies in NP, as selection evidently also occurred in hominids, and such deviation was not found in this lineage.

**Figure 7:**
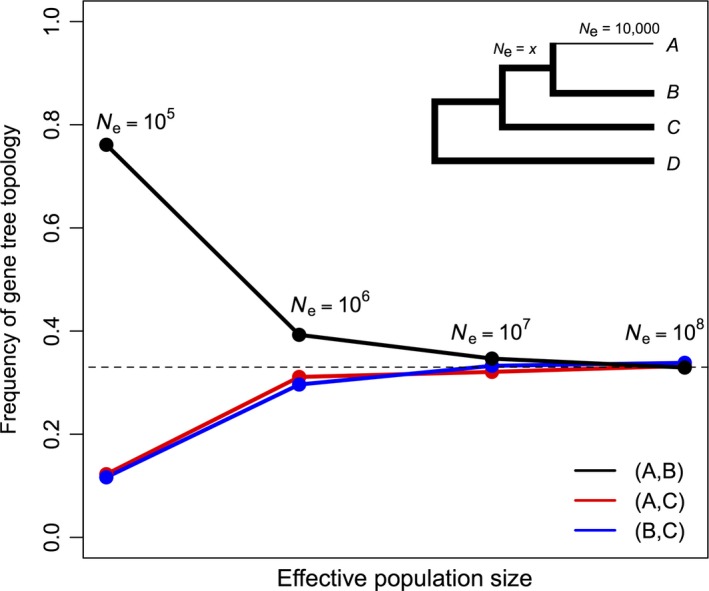
Simulation to evaluate the effect of long branch attraction in gene trees between lineages sharing large effective population sizes. Population size of lineage A was fixed at 10,000 Wright‐Fisher individuals, while lineages shown in thick branches had population size *x*, which varied from 10^5^ to 10^8^ W‐F individuals. Node ages were set at the values calculated for NP by Schrago et al. (2014). A total of ten alleles were simulated in each branch using the MCcoal program of the BPP 3.3 package (Yang, 2015)

It may be argued that demographic factors also impact the standard MSC model, as lineages with large effective population sizes will tend to be clustered in gene trees due to a higher chance of sharing ancestral polymorphisms. To investigate this hypothesis, we simulated alleles in a four‐species phylogeny with increasing difference between the effective population sizes of the two lineages composing the most inclusive clade (Figure 7). Irrespective of the variation in effective population sizes inputted, the distribution of mismatch gene trees followed the MSC model. Therefore, as population sizes increased, all three possible gene tree topologies tended to be equally likely, exactly as expected by the standard equations for three species rooted by an outgroup (Pamilo & Nei, 1988). We thus suggest that ancestral demographic dynamics fail to adequately explain our results for NP.

In conclusion, by comparing anthropoid primate datasets with significantly different rates of karyotype evolution, we found that predictions of the MSC theory under a four‐species case were not fulfilled in NP, presumably because of substantial chromosomal shuffling. We also concluded that, despite significant departure from MSC in NP, the topological inference of the species tree using heuristic MSC methods worked well independently of sequence length. It was harder for MSC theory to provide consistent estimates of population‐level parameters. Estimates of ancestral branch lengths in coalescent units may vary across the genome because the effective population size is influenced by the action of selection and recombination (Charlesworth, 2002, 2009). This substantial variation of estimates of coalescent‐related parameters using fundamentally the same dataset is problematic, and further developments should address this discrepancy, possibly by accommodating the uncertainty of gene tree topologies in parametric inference. The standard practice of concatenating sequences was also successful in resolving the difficult problem of the phylogeny of the major NP lineages, which consisted of rapid speciations associated with large ancestral effective population size (Schrago et al., 2014). Finally, we suggest that the distribution of adjacent PI sites along the genome is useful to measure the level of chromosomal shuffling along the evolution of a lineage.

## CONFLICT OF INTEREST

The authors declare that they have no conflict of interest.

## AUTHOR CONTRIBUTIONS

CGS, BM, and HNS designed the experiments, conducted analyses, and wrote the manuscript. AGP and CF conducted analyses.

## References

[ece33736-bib-0002] Anisimova, M. , & Gascuel, O. (2006). Approximate likelihood‐ratio test for branches: A fast, accurate, and powerful alternative. Systematic Biology, 55, 539–552. https://doi.org/10.1080/10635150600755453 16785212 10.1080/10635150600755453

[ece33736-bib-0003] Bonvicino, C. R. , Penna‐Firme, V. , do Nascimento, F. F. , Lemos, B. , Stanyon, R. , & Seuanez, H. N. (2003). The lowest diploid number (2n=16) yet found in any primate: Callicebus lugens (Humboldt, 1811). Folia Primatologica, 74, 141–149. https://doi.org/10.1159/000070647 10.1159/000070647 12826733

[ece33736-bib-0004] Burgess, R. , & Yang, Z. (2008). Estimation of hominoid ancestral population sizes under Bayesian coalescent models incorporating mutation rate variation and sequencing errors. Molecular Biology and Evolution, 25, 1979–1994. https://doi.org/10.1093/molbev/msn148 18603620 10.1093/molbev/msn148

[ece33736-bib-0005] Charlesworth, B. (2002). Effective population size. Current Biology, 12, R716–R717. https://doi.org/10.1016/S0960-9822(02)01244-7 10.1016/s0960-9822(02)01244-7 12419196

[ece33736-bib-0006] Charlesworth, B. (2009). Fundamental concepts in genetics: Effective population size and patterns of molecular evolution and variation. Nature Reviews Genetics, 10, 195–205. https://doi.org/10.1038/nrg2526 10.1038/nrg2526 19204717

[ece33736-bib-0007] Chen, F. C. , & Li, W. H. (2001). Genomic divergences between humans and other hominoids and the effective population size of the common ancestor of humans and chimpanzees. American Journal of Human Genetics, 68, 444–456. https://doi.org/10.1086/318206 11170892 10.1086/318206 PMC1235277

[ece33736-bib-0008] Degnan, J. H. , & Rosenberg, N. A. (2009). Gene tree discordance, phylogenetic inference and the multispecies coalescent. Trends in Ecology & Evolution, 24, 332–340. https://doi.org/10.1016/j.tree.2009.01.009 19307040 10.1016/j.tree.2009.01.009

[ece33736-bib-0009] Degnan, J. H. , & Salter, L. A. (2005). Gene tree distributions under the coalescent process. Evolution, 59, 24–37. https://doi.org/10.1111/j.0014-3820.2005.tb00891.x 15792224

[ece33736-bib-0010] Edwards, S. V. (2009). Is a new and general theory of molecular systematics emerging? Evolution, 63, 1–19. https://doi.org/10.1111/j.1558-5646.2008.00549.x 19146594 10.1111/j.1558-5646.2008.00549.x

[ece33736-bib-0011] Edwards, S. V. , Xi, Z. X. , Janke, A. , Faircloth, B. C. , McCormack, J. E. , Glenn, T. C. , … Davis, C. C. (2016). Implementing and testing the multispecies coalescent model: A valuable paradigm for phylogenomics. Molecular Phylogenetics and Evolution, 94, 447–462. https://doi.org/10.1016/j.ympev.2015.10.027 26518740 10.1016/j.ympev.2015.10.027

[ece33736-bib-0014] Guindon, S. , Dufayard, J.‐F. , Lefort, V. , Anisimova, M. , Hordijk, W. , & Gascuel, O. (2010). New algorithms and methods to estimate maximum‐likelihood phylogenies: Assessing the performance of PhyML 3.0. Systematic Biology, 59, 307–321. https://doi.org/10.1093/sysbio/syq010 20525638 10.1093/sysbio/syq010

[ece33736-bib-0015] Hobolth, A. , Christensen, O. F. , Mailund, T. , & Schierup, M. H. (2007). Genomic relationships and speciation times of human, chimpanzee, and gorilla inferred from a coalescent hidden Markov model. Plos Genetics, 3, 294–304. 10.1371/journal.pgen.0030007 PMC1802818 17319744

[ece33736-bib-0016] Jarvis, E. D. , Mirarab, S. , Aberer, A. J. , Li, B. , Houde, P. , Li, C. , … Zhang, G. (2014). Whole‐genome analyses resolve early branches in the tree of life of modern birds. Science, 346, 1320–1331. https://doi.org/10.1126/science.1253451 25504713 10.1126/science.1253451 PMC4405904

[ece33736-bib-0017] Lanier, H. C. , & Knowles, L. L. (2012). Is recombination a problem for species‐tree analyses? Systematic Biology, 61, 691–701. https://doi.org/10.1093/sysbio/syr128 22215721 10.1093/sysbio/syr128

[ece33736-bib-0018] Liu, L. , Xi, Z. , & Davis, C. C. (2015). Coalescent methods are robust to the simultaneous effects of long branches and incomplete lineage sorting. Molecular Biology and Evolution, 32, 791–805. https://doi.org/10.1093/molbev/msu331 25431481 10.1093/molbev/msu331

[ece33736-bib-0019] Liu, L. , Yu, L. , & Edwards, S. V. (2010). A maximum pseudo‐likelihood approach for estimating species trees under the coalescent model. BMC Evolutionary Biology, 10, 302. 20937096 10.1186/1471-2148-10-302 PMC2976751

[ece33736-bib-0020] Liu, L. , Yu, L. , Kubatko, L. , Pearl, D. K. , & Edwards, S. V. (2009a). Coalescent methods for estimating phylogenetic trees. Molecular Phylogenetics and Evolution, 53, 320–328. https://doi.org/10.1016/j.ympev.2009.05.033 19501178 10.1016/j.ympev.2009.05.033

[ece33736-bib-0022] Liu, L. , Yu, L. L. , Pearl, D. K. , & Edwards, S. V. (2009b). Estimating species phylogenies using coalescence times among sequences. Systematic Biology, 58, 468–477. https://doi.org/10.1093/sysbio/syp031 20525601 10.1093/sysbio/syp031

[ece33736-bib-0023] Lynch, M. (2007). The frailty of adaptive hypotheses for the origins of organismal complexity. Proceedings of the National Academy of Sciences of the United States of America, 104, 8597–8604. https://doi.org/10.1073/pnas.0702207104 17494740 10.1073/pnas.0702207104 PMC1876435

[ece33736-bib-0024] Maddison, W. P. (1997). Gene trees in species trees. Systematic Biology, 46, 523–536. https://doi.org/10.1093/sysbio/46.3.523

[ece33736-bib-0027] McCormack, J. E. , Faircloth, B. C. , Crawford, N. G. , Adair Gowaty, P. , Brumfield, R. T. , & Glenn, T. C. (2012). Ultraconserved elements are novel phylogenomic markers that resolve placental mammal phylogeny when combined with species‐tree analysis. Genome Research, 22, 746–754. https://doi.org/10.1101/gr.125864.111 22207614 10.1101/gr.125864.111 PMC3317156

[ece33736-bib-0028] Mirarab, S. , Reaz, R. , Bayzid, M. S. , Zimmermann, T. , Swenson, M. S. , & Warnow, T. (2014). ASTRAL: Genome‐scale coalescent‐based species tree estimation. Bioinformatics, 30, I541–I548. https://doi.org/10.1093/bioinformatics/btu462 25161245 10.1093/bioinformatics/btu462 PMC4147915

[ece33736-bib-0029] de Oliveira, E. H. , Neusser, M. , Pieczarka, J. C. , Nagamachi, C. , Sbalqueiro, I. J. , & Muller, S. (2005). Phylogenetic inferences of Atelinae (Platyrrhini) based on multi‐directional chromosome painting in Brachyteles arachnoides, Ateles paniscus paniscus and Ateles b. marginatus. Cytogenetic and Genome Research, 108, 183–190. https://doi.org/10.1159/000080814 15545728 10.1159/000080814

[ece33736-bib-0030] Pamilo, P. , & Nei, M. (1988). Relationship between gene trees and species trees. Molecular Biology and Evolution, 5, 568–583. 3193878 10.1093/oxfordjournals.molbev.a040517

[ece33736-bib-0031] Perelman, P. , Johnson, W. E. , Roos, C. , Seuanez, H. N. , Horvath, J. E. , Moreira, M. A. , … Pecon‐Slattery, J. (2011). A molecular phylogeny of living primates. Plos Genetics, 7, e1001342 https://doi.org/10.1371/journal.pgen.1001342 21436896 10.1371/journal.pgen.1001342 PMC3060065

[ece33736-bib-0032] Ronquist, F. , & Huelsenbeck, J. P. (2003). MrBayes 3: Bayesian phylogenetic inference under mixed models. Bioinformatics, 19, 1572–1574. https://doi.org/10.1093/bioinformatics/btg180 12912839 10.1093/bioinformatics/btg180

[ece33736-bib-0033] Schneider, H. , Schneider, M. P. , Sampaio, I. , Harada, M. L. , Stanhope, M. , Czelusniak, J. , & Goodman, M. (1993). Molecular phylogeny of the New World monkeys (Platyrrhini, primates). Molecular Phylogenetics and Evolution, 2, 225–242. https://doi.org/10.1006/mpev.1993.1022 8136923 10.1006/mpev.1993.1022

[ece33736-bib-0034] Schrago, C. G. (2007). On the time scale of new world primate diversification. American Journal of Physical Anthropology, 132, 344–354. https://doi.org/10.1002/(ISSN)1096-8644 17133436 10.1002/ajpa.20459

[ece33736-bib-0035] Schrago, C. G. (2014a). The effective population sizes of the anthropoid ancestors of the human‐chimpanzee lineage provide insights on the historical biogeography of the great apes. Molecular Biology and Evolution, 31, 37–47. https://doi.org/10.1093/molbev/mst191 24124206 10.1093/molbev/mst191

[ece33736-bib-0036] Schrago, C. G. (2014b). The limiting distribution of the effective population size of the ancestor of humans and chimpanzees. Journal of Theoretical Biology, 357, 55–61. https://doi.org/10.1016/j.jtbi.2014.05.009 24834834 10.1016/j.jtbi.2014.05.009

[ece33736-bib-0037] Schrago, C. G. , Menezes, A. N. , Furtado, C. , Bonvicino, C. R. , & Seuanez, H. N. (2014). Multispecies coalescent analysis of the early diversification of neotropical primates: Phylogenetic inference under strong gene trees/species tree conflict. Genome Biology and Evolution, 6, 3105–3114. https://doi.org/10.1093/gbe/evu244 25377940 10.1093/gbe/evu244 PMC4255775

[ece33736-bib-0038] Seuanez, H. N. (1979). The phylogeny of human chromosomes. Berlin, Heidelberg, New York: Springer‐Verlag https://doi.org/10.1007/978-3-642-67260-6

[ece33736-bib-0040] Sherlock, J. K. , Griffin, D. K. , Delhanty, J. D. A. , & Parrington, J. M. (1996). Homologies between human and marmoset (*Callithrix jacchus*) chromosomes revealed by comparative chromosome painting. Genomics, 33, 214–219. https://doi.org/10.1006/geno.1996.0186 8660970 10.1006/geno.1996.0186

[ece33736-bib-0041] Song, S. , Liu, L. , Edwards, S. V. , & Wu, S. (2012). Resolving conflict in eutherian mammal phylogeny using phylogenomics and the multispecies coalescent model. Proceedings of the National Academy of Sciences of the United States of America, 109, 14942–14947. https://doi.org/10.1073/pnas.1211733109 22930817 10.1073/pnas.1211733109 PMC3443116

[ece33736-bib-0042] Springer, M. S. , & Gatesy, J. (2016). The gene tree delusion. Molecular Phylogenetics and Evolution, 94, 1–33. https://doi.org/10.1016/j.ympev.2015.07.018 26238460 10.1016/j.ympev.2015.07.018

[ece33736-bib-0043] Stanyon, R. , Bonvicino, C. R. , Svartman, M. , & Seuanez, H. N. (2003). Chromosome painting in *Callicebus lugens*, the species with the lowest diploid number (2n=16) known in primates. Chromosoma, 112, 201–206. https://doi.org/10.1007/s00412-003-0261-5 14608465 10.1007/s00412-003-0261-5

[ece33736-bib-0400] Stanyon, R. , Rocchi, M. , Capozzi, O. , Roberto, R. , Misceo, D. , Ventura M. , Cardone M. F. , Bigoni F. , Archidiacono N . (2008). Primate chromosome evolution: ancestral karyotypes, marker order and neocentromeres. Chromosome Research, 16, 17–39. https://doi:10.1007/s10577-007-1209-z. 18293103 10.1007/s10577-007-1209-z

[ece33736-bib-0045] Wildman, D. E. , Jameson, N. M. , Opazo, J. C. , & Yi, S. V. (2009). A fully resolved genus level phylogeny of neotropical primates (Platyrrhini). Molecular Phylogenetics and Evolution, 53, 694–702. https://doi.org/10.1016/j.ympev.2009.07.019 19632342 10.1016/j.ympev.2009.07.019

[ece33736-bib-0046] Yang, Z. (2015). A tutorial of BPP for species tree estimation and species delimitation. Current Zoology, 61, 854–865. https://doi.org/10.1093/czoolo/61.5.854

[ece33736-bib-0047] Yunis, J. J. , & Prakash, O. (1982). The origin of man: A chromosomal pictorial legacy. Science, 215, 1525–1530. https://doi.org/10.1126/science.7063861 7063861 10.1126/science.7063861

